# Anti-MDA5 Positive Juvenile Dermatomyositis With Macrophage Activation Syndrome: A Case Report

**DOI:** 10.7759/cureus.75137

**Published:** 2024-12-05

**Authors:** Alaa S Mehair, Miroslav Harjacek

**Affiliations:** 1 Pediatric Medicine, Tawam Hospital, Abu Dhabi, ARE

**Keywords:** anti- melanoma differentiation-associated protein 5 antibody, hyperferritinemia, inflammatory, inflammatory myopathies, juvenile dermatomyositis, macrophage activation syndrome

## Abstract

There are several types of inflammatory myopathies, including juvenile dermatomyositis (JDM), which is characterized by muscle inflammation that can eventually lead to weakness. A devastating complication of JDM is macrophage activation syndrome (MAS), although reports of MAS in JDM patients are limited. Additionally, cases of JDM associated with positive anti-MDA5 are rare and represent a fatal subtype of inflammatory myopathies, with a significant risk of lung impairment. This case report discusses a six-year-old girl presenting with proximal muscle weakness and cutaneous changes, including heliotrope rash and Gottron’s papules, who was diagnosed with JDM. Muscle involvement was confirmed through MRI of the thigh muscles. Laboratory findings revealed elevated aldolase, hepatitis, high lactate dehydrogenase, and hyperferritinemia, leading to a diagnosis of JDM complicated by MAS. Pulse steroid therapy combined with cyclosporine was initiated, and she showed significant improvement initially. However, she later tested positive for anti-MDA5 antibodies, a marker associated with a poor prognosis. Highlighting MAS as a potential complication in JDM cases with positive anti-MDA5 antibodies can help physicians recognize this outcome and consider it in the differential diagnosis.

## Introduction

Inflammatory myopathies encompass several subtypes, including juvenile dermatomyositis (JDM), which is characterized by muscle inflammation that can lead to weakness [1]. The hallmark signs of JDM include distinctive skin manifestations such as heliotrope rash and Gottron’s papules. The presence of these skin findings, combined with symmetrical proximal muscle weakness, should raise suspicion for JDM [2].

Anti-MDA5 is a myositis-specific antibody, and the presence of anti-MDA5 in dermatomyositis is a subtype that is associated with distinctive skin lesions and an increased risk of rapidly progressive interstitial lung disease [3].

Macrophage activation syndrome (MAS) is a severe complication that can occur in various rheumatological conditions. However, studies examining the co-occurrence of anti-MDA5 dermatomyositis and MAS are limited, necessitating further investigation. This report aims to increase awareness among physicians about similar cases.

## Case presentation

A six-year-old girl was admitted to our hospital with generalized muscle weakness, fatigue, and subjective fever lasting for two months. Seven months prior to admission, she began experiencing pain in her wrists, elbows, knees, and back, followed by complaints of vague, generalized headaches. Over this period, she also lost approximately 7 kg. Additionally, she noticed changes in her voice and skin, primarily affecting her face and hands. She was seen by a physician in her homeland, who suggested the possibility of a rheumatological disease. However, no laboratory investigations were conducted due to limited resources. Although no specific diagnosis was made, she was prescribed prednisolone (2 mg), hydroxychloroquine (20 mg), and azathioprine, which she took for two months before switching to leflunomide. Her family noted that, since starting these medications, she became more fatigued and started losing hair. Over time, her gait deteriorated, and she became unable to perform daily activities, such as getting out of bed, combing her hair, or dressing. For the past two months, she experienced daily subjective fevers without chills or night sweats. She denied photosensitivity or personality changes. Before this, she had been healthy and performed well at school. A complete review of her medical history revealed a family history of an aunt with rheumatoid arthritis.

**Figure 1:**
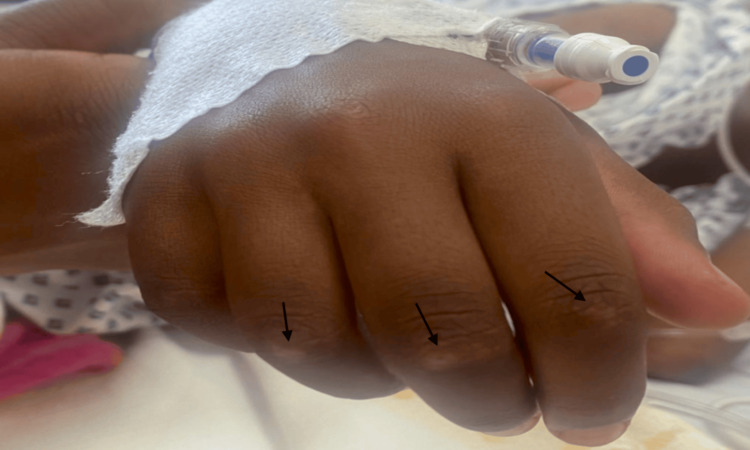
Gottron’s papules over the interphalangeal joints of the fingers

Upon admission to our hospital, she was febrile (38.8°C), while other vital signs were within normal limits. She appeared ill and cachectic, with a BMI of 12.68 kg/m². Characteristic cutaneous changes were observed, including heliotrope rash and Gottron’s papules over the interphalangeal joints (Figure 1).

**Figure 2:**
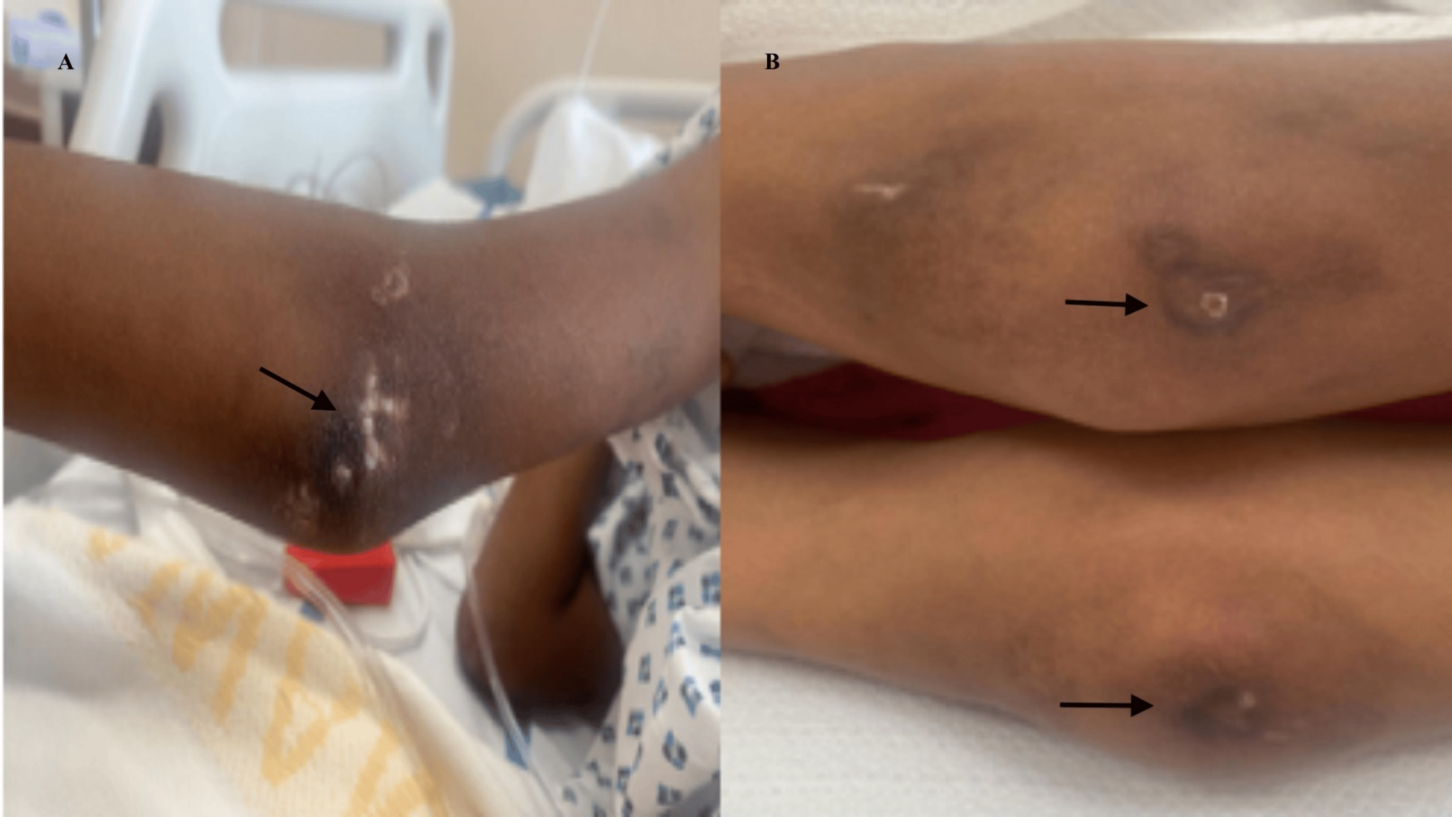
Skin ulcerations and cutis calcinosis (A) Cutis calcinosis over the left elbow. (B) Skin ulceration on both knees.

Skin ulcerations and cutis calcinosis were noted over the sacrum, knees, and elbows (Figure 2).

Decreased muscle strength was detected symmetrically in the proximal segments of both the upper (Medical Research Council (MRC) Scale for muscle strength grade 1) and lower extremities (MRC grade 2).

Laboratory investigations revealed elevated levels of ferritin, lactate dehydrogenase, fibrinogen, ESR, and pancytopenia (Table 1).

**Table 1:** Laboratory investigations

Lab investigations	Value	Reference range
White blood cell count	4.4 × 10^9^/L (Low)	5-14.5 × 10^9^/L
Neutrophils count	3.14 × 10^9^/L	1.5-8.5 × 10^9^/L
Red blood cell count	97 g/L (Low)	11.5-14.5 g/L
Platelets count	121 × 10^9^/L (Low)	140-400 × 10^9^/L
Ferritin level	1,074 mcg/L (High)	7-84 mcg/L
Iron level	3.7 micromol/L (Low)	5.8-34.5 micromol/L
Lactate dehydrogenase	600 IU/L (High)	<2.59 IU/L
Triglycerides	1.95 mmol/L (High)	0.25-0.85 mmol/L
Fibrinogen	2.22 g/L	1.5-3.87 g/L
Creatine kinase	153 IU/L	26-192 IU/L
Creatine kinase MB	1.2 mcg/L	<3.6 mcg/L
Alanine aminotransferase	370 IU/L (High)	<19 IU/L
Aspartate transferase	791 IU/L (High)	<41 IU/L
Aldolase	28.42 U/L (High)	<15.00 U/L
Antinuclear antibodies	Positive titer: 1:320	-
Anti-double stranded DNA	Negative: 20.2 IU/mL	-
C3 complement	0.92 g/L (Negative)	0.9-1.8 g/L
C4 complement	0.21 g/L (Negative)	0.1-0.4 g/L
Rheumatoid factor	Negative	-
Erythrocyte sedimentation rate	39 mm/hr (High)	<20 mm/hr
Procalcitonin	0.34 ng/mL	<0.1 ng/mL

**Figure 3:**
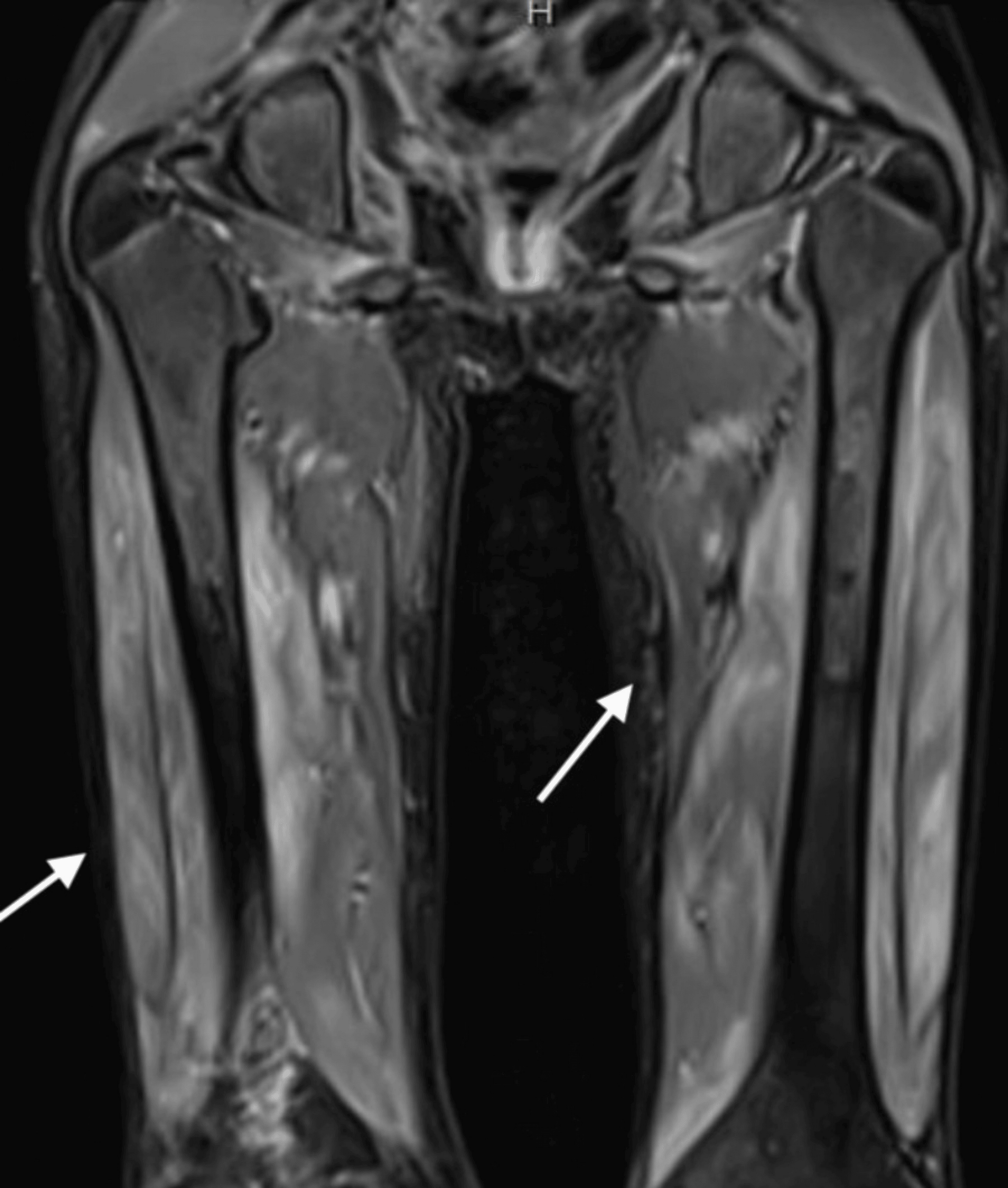
MRI of the thigh showing enhancement of the subcutaneous tissue after contrast administration

The creatine kinase level was normal, but aldolase was nearly double the normal value. Blood and urine cultures were negative. A baseline cardiac echocardiogram showed normal heart function, with trivial, clinically insignificant mitral regurgitation. An MRI of the thighs with contrast revealed diffuse inflammatory changes involving the quadriceps and, to a lesser extent, the hamstrings/adductors bilaterally (Figure 3).

A bone marrow biopsy was also performed and returned normal. Given the clinical presentation, laboratory findings, and MRI results, which were diagnostic for JDM with MAS, no muscle biopsy was performed. The patient was treated immediately with 3 days of pulse methylprednisolone (30 mg/kg), followed by a continuation of prednisolone (2 mg/kg) and cyclosporine (3 mg/kg). Clinical improvement was observed, with increased muscle strength, as she was able to walk independently. Serial laboratory investigations showed improvement in inflammatory markers after the initiation of pulse steroid therapy (Table 2).

**Table 2:** Post-steroid laboratory results

Post-steroid days	Laboratory levels				
	Ferritin (mcg/L)	Triglycerides (mmol/L)	Platelet (× 10^9^/L)	White blood cells (× 10^9^/L)	Fibrinogen (g/L)
Day 1	1256	1.95	101	3.7	1.91
Day 3	763	1.58	118	3.2	1.93
Day 7	625	5.69	129	4.6	1.69
Day 10	432	3.66	182	6.3	1.28
Day 14	356	2.86	199	6.8	1.32

**Figure 4:**
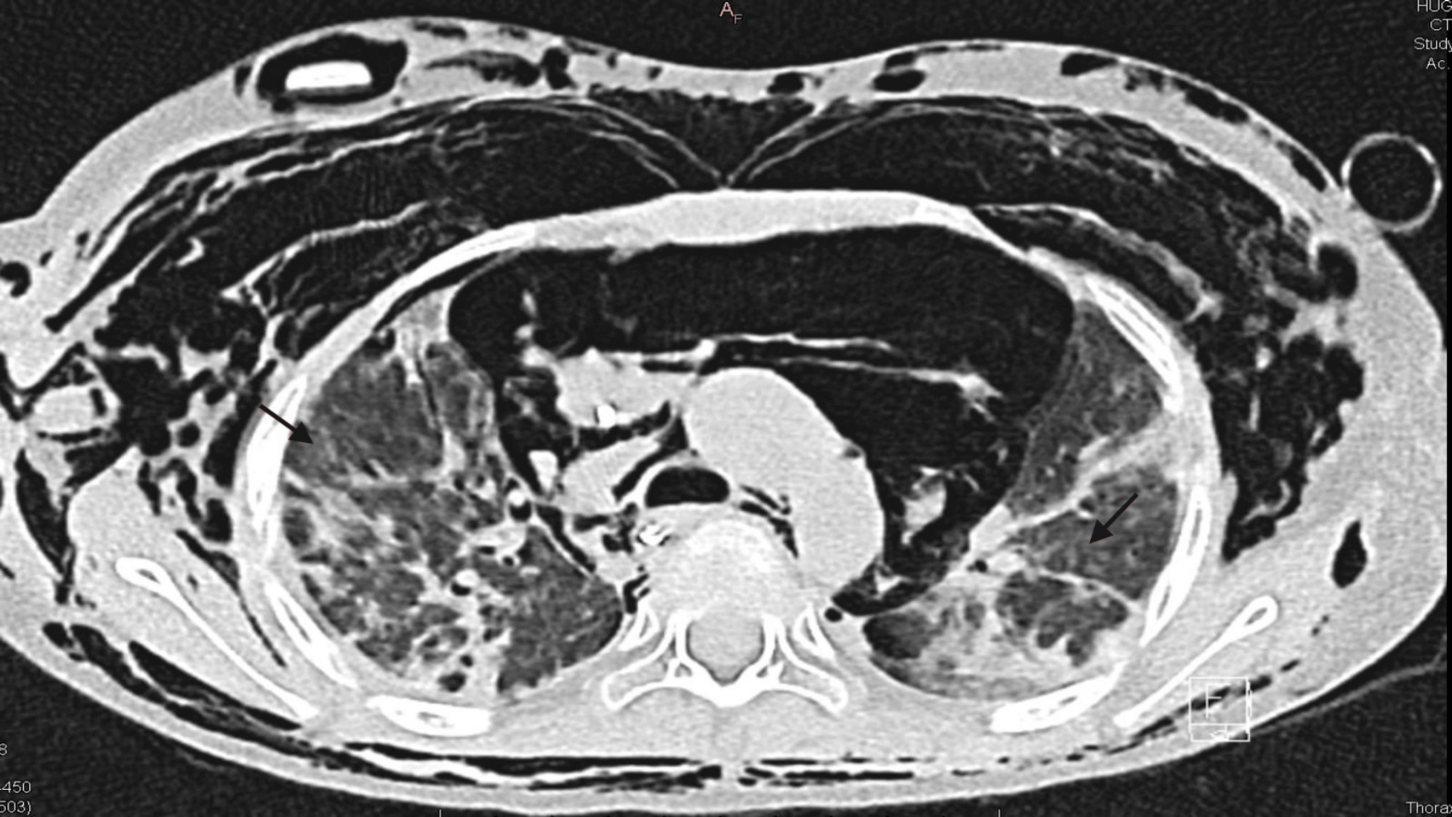
Chest CT scan showing fibrotic changes in the lungs

During subsequent follow-up, additional markers for the diagnosis of JDM were tested, and she was found to be positive for anti-MDA5. However, she tested negative for anti-Mi2 alpha, anti-Mi2 beta, and anti-TIF1 gamma. After discharge from our hospital, she developed breathing difficulties and was eventually admitted to an intensive care unit abroad due to the need for invasive ventilation in the setting of hypoxia. A chest CT scan revealed fibrotic changes in the lungs (Figure 4).

## Discussion

This case reports anti-MDA5-positive JDM with concomitant MAS, confirmed through clinical symptoms and laboratory findings. To date, approximately 18 cases of JDM with MAS have been reported. According to a study by Chang et al., most patients developed MAS within three months of JDM diagnosis [1].

Our patient tested positive for anti-MDA5, which is considered a poor prognostic marker. anti-MDA5-positive JDM is a subtype of dermatomyositis characterized by distinctive skin lesions and an increased risk of rapidly progressive interstitial lung disease. Early detection of similar cases is crucial for improving outcomes, as delayed management can lead to a poorer prognosis, as highlighted by Otha et al. [4].

One of the challenges in managing anti-MDA5-positive JDM is the limited treatment options, as it is a rare condition compared to other rheumatological diseases. Some studies suggest the use of JAK inhibitors targeting the IFN-1 pathway [5,6]. However, further research and established guidelines are needed for managing this specific condition.

In an adult review article, Nombel et al. reported that the prevalence of anti-MDA5-positive dermatomyositis ranges from 7% to 60%, with a higher prevalence in Asians (11-60%) compared to Caucasians (7-16%). Similar to other autoimmune diseases, anti-MDA5-positive dermatomyositis predominantly affects women, with a female-to-male ratio ranging from 0.6 to 7.3. Although the pathogenic mechanisms remain poorly understood, they are believed to result from a specific gene-environment interaction [7].

Given the potentially fatal complications associated with anti-MDA5-positive JDM, testing for anti-MDA5 should be performed as soon as the diagnosis of JDM is made. Similar to the findings reported by Yeung et al., lung fibrosis was observed in this case as a sequela of the condition [5]. Furthermore, performing a chest X-ray early, even in the absence of clinical respiratory symptoms, can help identify the underlying subtype of JDM.

## Conclusions

JDM cases with positive anti-MDA5 are rare and represent a fatal subtype of inflammatory myopathies, with a significant risk of lung impairment. While MAS is not typically considered a common complication of JDM, its prevalence may be underreported. Therefore, raising awareness of MAS as a potential complication is crucial for physicians to consider it in their differential diagnosis.

This case underscores the importance of recognizing MAS in patients diagnosed with anti-MDA5-positive JDM, and highlights the need for greater global awareness in approaching similar cases.
